# Intraclass correlation coefficients for cluster randomized trials in care pathways and usual care: hospital treatment for heart failure

**DOI:** 10.1186/1472-6963-14-84

**Published:** 2014-02-24

**Authors:** Seval Kul, Kris Vanhaecht, Massimiliano Panella

**Affiliations:** 1 Department of Biostatistics, Faculty of Medicine, University of Gaziantep, Gaziantep, Turkey; 2 Department of Public Health and Primary Care, KULeuven, University of Leuven, and University Hospitals Leuven, Leuven, Belgium; 3 Western Norway Network on Integrated Care, Helse Fonna, Haugesund, Norway; 4 European Pathway Association, Leuven, Belgium; 5 Department of Clinical and Experimental Medicine, University of Eastern Piedmont 'A. Avogadro', Novara, Italy

**Keywords:** Care pathways, Heart failure, Intraclass correlation coefficient, Multicenter cluster randomized trials

## Abstract

**Background:**

Cluster randomized trials are increasingly being used in healthcare evaluation to show the effectiveness of a specific intervention. Care pathways (CPs) are becoming a popular tool to improve the quality of health-care services provided to heart failure patients. In order to perform a well-designed cluster randomized trial to demonstrate the effectiveness of Usual care (UC) and CP in heart failure treatment, the intraclass correlation coefficient (ICC) should be available before conducting a trial to estimate the required sample size. This study reports ICCs for both demographical and outcome variables from cluster randomized trials of heart failure patients in UC and care pathways.

**Methods:**

To calculate the degree of within-cluster dependence, the ICC and associated 95% confidence interval were calculated by a method based on analysis of variance. All analyses were performed in R software version 2.15.1.

**Results:**

ICCs for baseline characteristics ranged from 0.025 to 0.058. The median value and interquartile range was 0.043 [0.026-0.052] for ICCs of baseline characteristics. Among baseline characteristics, the highest ICCs were found for admission by referral or admission from home (ICC = 0.058) and the disease severity at admission (ICC = 0.046). Corresponding ICCs for appropriateness of the stay, length of stay and hospitalization cost were 0.069, 0.063, and 0.001 in CP group and 0.203, 0.020, 0.046 for usual care, respectively.

**Conclusion:**

Reported values of ICCs from present care pathway trial and UC results for some common outcomes will be helpful for estimating sample size in future clustered randomized heart failure trials, in particular for the evaluation of care pathways.

## Background

Cluster randomized trials (CRTs) are increasingly used to evaluate the effectiveness of health-care interventions [1]. In CRTs, patients are nested within clusters such as hospitals, communities or practices, and interventions are applied at cluster levels but outcomes are measured at the individual level. It is expected that individuals in the same cluster, e.g. geographical area, hospital, would have more similarities compared to individuals in different clusters [2]. This similarity may occur because patients in the same clusters interact with each other and receive care from the same practitioners. Intraclass correlation coefficient (ICC) is used to determine the degree of within-cluster dependence and it plays an important role in estimating sample size for cluster randomized trials [3].

In cluster randomised trials, traditional sample size estimation techniques for RCTs lead to underestimation of the required sample size [3,4] and because individuals in the same organization or clusters are not independent, the sample size must be inflated [4]. The degree of the increase in sample size is a function of both ICC and cluster sizes where generally a greater ICC requires enrollment of a greater number of patients in the trial [3].

Chronic heart failure (CHF) is a major health problem worldwide associated with a high prevalence, mortality rate and hospital costs. It is estimated that about 5.7 million people are afflicted by CHF in the United States [5] and in Europe approximately 5% of all acute medical admissions are HF-related [6]. The estimated direct and indirect costs of HF management in the United States totaled $39.2 billion in 2010 [7]. It was reported that the cost of HF care is two times higher than that of breast cancer, and three times higher compared to costs of colorectal and lymphoma cancer care in the USA [8].

Care Pathway (CP) is a complex intervention for the mutual decision making and organisation of care processes for a well-defined group of patients during a well-defined period [9]. CPs are also known as “integrated care pathways”, “critical pathways”, “care plans”, “clinical pathways”, “care maps” and “care protocols” [10]. CPs have become a popular tool to improve quality of health-care services provided to CHF patients by reducing the risks of mortality [11-13] and readmission [14,15] leading to shorter length of stay [13,14] and lower costs [13,14]. CPs are utilized to evaluate one specific care plan. Each hospital included in CPs uses the same protocol in practice. Many articles are available in Medline and medical databases describing the concept and success of CPs [9-11]. According to the European Care Pathway Association (an international non-profit association), the defining characteristics of CPs include: (i) an explicit statement of the goals and key elements of care, based on evidence, best practice, and patients’ expectations and their characteristics; (ii) the facilitation of the communication among the team members and with patients and families; (iii) the coordination of the care process by coordinating the roles and sequencing the activities of the multidisciplinary care team, patients and their relatives; (iv) the documentation, monitoring, and evaluation of variances and outcomes; and (v) the identification of the appropriate resources. The aim of a care pathway is to enhance the quality of care, across the continuum, by improving risk-adjusted patient outcomes, promoting patient safety, increasing patient satisfaction, and optimizing the use of resources [16].

In order to conduct a well-designed CRT with the aim to show the effectiveness of CPs for a CHF treatment, ideally, the ICC should be known beforehand to estimate required sample size and statistical power to decrease the chances of type II error [17]. A range of ICCs has been reported previously [17-22]; however, it is often difficult to obtain an appropriate ICC value for a specific study from the published ICC estimates. The present study reports ICCs for both demographical and outcome variables from cluster randomized trials of heart failure patients in the setting of usual care (UC) and care pathways (CP). Reported values of ICCs obtained from the UC and care pathway trials for some common outcomes would be useful to estimate sample size in future CP trials.

## Methods

### Experimental design

The intraclass correlation coefficients and confidence intervals for outcome variables of interest were calculated from a multi-center CRT which assessed in-hospital treatment of heart failure. In the present study, 14 community hospitals were randomized either to care pathway or usual care. Data were collected from March 2003 to October 2004 prospectively by trained physicians and nurses.

All patients with a primary diagnosis of HF who received in-hospital treatment and patients with acute myocardial infarction or unstable angina were enrolled. One physician or nurse with at least 2 years of experience in CP was assigned to each hospital in the experimental group. The final sample consisted of 429 patients (CP, n = 214 and UC, n = 215). The trial was successful in reducing in-hospital mortality and unscheduled readmissions in the care pathway group.

More details about the study protocol and intervention have been previously described elsewhere [13,23].

### Ethics

The project was exempt from ethical clearance according to the Italian Ministry of Health law number (ex art. 12bis D.lgs 229/99). Moreover the aim of the study is to improve quality of care through clinical pathways and thus should not imply any risk for the patients affected by the study. It is difficult to imagine that our intervention based on better evidences and appropriate use of technologies and drugs could worsen the quality of care when compared to usual care. So according to other experiences dealing with clinical pathways or implementation of evidence based guidelines in practice we think that a Committee of Research Ethic would not consider it necessary to submit the protocol for approval.

### Statistical method

To calculate the degree of within-cluster dependence, the ICCs for continuous variables and ordinal variables were calculated by the formula which was derived by Donner and Klar based on an analysis of variance [24];

$$\mathrm{ICC}=\frac{S_{b}^{2}}{\left(S_{b}^{2}+S_{w}^{2}\right)}$$

where:

$s_{b}^{2}$ is the variance between clusters, and $s_{w}^{2}$ is the variance within clusters.

The confidence interval estimates for continuous variables and ordinal variables were calculated using the approximate formulas for the standard error of the ICC estimate [25]. Point estimates of the ICC from clustered binomial data were calculated using the logistic binomial-Gaussian model [26] and 500 replicates boostrap confidence interval estimations were presented to increase the precision of the estimates [27].

Design effect is the ratio between the number of subjects in the cluster study and the number of subjects in an equally reliable, randomly sampled unclustered study [28]. It is defined as the ratio of two variances: the variance of the estimator when the effect of clustering is taken into account over the variance of the estimator under the hypothesis of a simple random sample [3]. The design effect is estimated by using the following formula: Design effect = 1+ [m-1]*ICC. m is the average number of the individuals in each cluster [3]. The design effect varies for each outcome. Negative ICCs were truncated at zero because it has been suggested that negative ICCs should not be used for sample size calculation in CRTs [2].

All analyses were performed using R software version x2.15.1. The ICCs for continuous and ordinal variables were calculated using multilevel package and ICC estimates for binary variables and boostrap confidence intervals were calculated using aod package in R.

## Results

ICCs and 95% confidence intervals (CIs) were calculated for 429 heart failure patients (CP, n = 214 and UC, n = 215). Data were collected from 14 hospitals. Cluster size ranged between 30 to 32 and average cluster size was 30.64. For ICC estimates of baseline characteristics, all centers was used because the intervention has not yet occurred. Baseline characteristics and outcome variables of the study are presented in Table 1 and Table 2 respectively. Mean age of the study participants was 81.66 ±8.41 years (range: 50–99) and 49.4% of them were males. The most common health problems among the study patients were hypertension (73.7%] and co-morbidities such as COPD, diabetes and smoking (32.9%]. 48.5% of the patients admitted following referral by a general practitioner.

**Table 1:** Baseline characteristics of the sample in clusters

**No**	**n**	Age* (years)	**Male**^**†**^	**NYHA**^**† **^**class at admission**			**HT**^**†**^	**CM**^**†**^
				**II**	**III**	**IV**		
				6.7	40.0	53.3		
CP 2	30	80.50 (8.32)	56.7	3.3	56.7	40.0	66.7	20.0
CP 3	31	79.39 (9.32)	48.4	12.9	58.1	29.0	71.0	29.0
CP 4	30	82.00 (7.49)	46.7	6.7	63.3	30.0	73.3	30.0
CP 5	31	82.13 (8.54)	41.9	3.2	71.0	25.8	64.5	29.0
CP 6	31	83.39 (7.32)	48.4	16.1	54.8	29.0	71.0	19.4
CP 7	31	82.29 (7.00)	51.6	3.2	41.9	54.8	80.6	29.0
UC 8	30	81.43 (8.61)	50.0	6.7	46.7	46.7	83.3	50.0
UC 9	30	78.77 (10.08)	53.3	3.3	50.0	46.7	83.3	43.3
UC10	32	78.94 (9.610)	46.9	0.0	56.3	43.8	71.9	43.8
UC11	31	76.23 (8.09)	51.6	6.5	51.6	41.9	74.2	38.7
UC12	30	80.53 (7.32)	53.3	6.7	40.0	53.3	86.7	43.3
UC13	30	82.60 (7.74)	40.0	10.0	50.0	40.0	86.7	40.0
UC 14	32	79.22 (6.48)	62.5	15.6	75.0	9.4	40.6	9.4
Total	429	80.66±8.41	49.4	7.2	54.1	38.7	73.7	32.9

CP: Care Pathways; UC: Usual Care; No: number of cluster; n: cluster size, COPD: Chronic obstructive pulmonary disease, NYHA: New York Heart Association; HT: hypertension; CM: comorbidity.

*Continuous variables (mean (SD)).

^†^Ordinal or Binary variable (%).

**Table 2:** Outcome variables of the present study in clusters

**No**	**n**	LOS* (days)	Cost (€)^*^	**NYHA**^**† **^**class at discharge**			**Mortality**^**†**^	**AOS**^**†**^	**UR**^**†**^
				**II**	**III**	**IV**			
CP 1	30	10.97 (5.44)	2227.30 (591.83)	42.9	53.6	3.6	6.7	96.7	6.7
CP 2	30	13.13 (5.94)	2171.77 (470.22)	39.3	57.1	3.6	3.3	73.3	0.0
CP 3	31	9.13 (4.33)	2077.87 (550.71)	48.3	51.7	0.0	6.5	77.4	9.7
CP 4	30	8.27 (4.82)	2113.47 (550.89)	46.4	50.0	3.6	6.7	73.3	13.3
CP 5	31	10.35 (4.72)	2089.52 (482.24)	31.0	65.5	3.4	6.5	58.1	6.5
CP 6	31	9.48 (4.86)	1969.10 (571.62)	50.0	46.7	3.3	3.2	64.5	16.1
CP 7	31	11.13 (4.98)	2234.29 (489.18)	31.0	65.5	3.4	6.5	90.3	3.2
Total CP	214	10.35 (5.17)	2125.56 (530.93)	41.3	55.7	3.0	5.6	76.2	7.9
UC 8	30	12.33 (7.63)	2207.53 (591.92)	24.0	64.0	12.0	16.7	83.3	13.3
UC 9	30	10.93 (6.11)	2322.50 (488.25)	45.5	40.9	13.6	20.0	76.7	10.0
UC10	32	10.13 (5.83)	2290.13 (525.57)	23.1	61.5	15.4	15.6	78.1	12.5
UC11	31	11.26 (6.00)	2243.74 (480.45)	11.1	74.1	14.8	12.9	77.4	16.1
UC12	30	9.43 (6.13)	2380.37 (719.91)	8.0	84.0	8.0	16.7	90.0	20.0
UC13	30	11.70 (6.82)	2150.43 (613.75)	25.9	66.7	7.4	10.0	76.7	23.3
UC 14	32	14.06 (7.65)	1898.31 (490.99)	77.8	22.2	0.0	15.6	25.0	3.1
Total UC	215	11.42 (6.69)	2211.21 (574.76)	30.7	59.2	10.1	15.3	72.1	14.0

No: number of cluster; n: cluster size; LOS: length of hospital stay; NYHA: New York Heart Association, AOS: Appropriateness of the stay; UR: Unscheduled readmission.

*Continuous variables (mean (SD)).

^†^Ordinal or Binary variable (%).

The mean length of hospital stay (LOS) for UC and CP patients was 11.42 ± 6.69 and 10.35 ± 5.17 days, with a mean cost of hospitalization of €2211.66 ± €574.76 and €2125,66 ± €530.93, respectively. In-hospital mortality rates were 15.3% in UC and 5.6% in the CP groups and the mean rates of unscheduled readmission for UC and CP were 14.0% and 7.9% respectively.

Tables 3 and 4 provide estimates of intraclass correlation coefficients, 95% confidence intervals and design effects for baseline characteristics for each cluster and outcomes of the clustered sample selected from 14 hospitals (7 CP, 7 UC).

**Table 3:** Intraclass correlation coefficients for baseline characteristics of the sample selected from 14 hospitals

**Variable**	**ICC**	**95% CI**
Age*	0.025	0.000-0.102
Disease Severity at Admission (NHYA)^†^	0.046	0.007-0.145
Admitted from Home or Referred by GP^†^	0.058	0.000-0.128
Co-morbidities^†^	0.027	0.000-0.066
Hypertension^†^	0.043	0.000-0.101

DE: Design effect, GP: General practitioner, COPD: Chronic obstructive pulmonary disease, NYHA: New York Heart Association; CI: confidence interval.

*Ordinal or Continuous variables.

^†^Binary variables.

**Table 4:** Intraclass correlation coefficients for the outcomes variables of study

	**Usual care**		**Care pathways**	
**Outcome variables**	**ICC**	**95% CI**	**ICC**	**95% CI**
LOS (days)*	0.020	0.000-0.184	0.063	0.007-0.311
Cost (€)*	0.046	0.001-0.265	0.001	0.000-0.107
In-hospital mortality^†^	0.001	0.000-0.003	0.001	0.000-0.003
Disease Severity at Discharge (NYHA)^†^	0.182	0.062-0.554	0.000	0.000-0.076
AOS^†^	0.203	0.059-0.436	0.069	0.003-0.155
Unscheduled readmission^†^	0.004	0.000-0.036	0.010	0.000-0.046

DE: Design effect, AOS: Appropriateness of the stay, NYHA: New York Heart Association, CI: confidence interval.

*Ordinal or Continuous variable.

^†^Binary variables.

ICCs for baseline characteristics ranged from 0.025 to 0.058. The median value and interquartile range for ICCs of baseline characteristics was 0.043 [0.026-0.052]. Among baseline characteristics, the highest ICCs were found for referrals by a general practitioner or admission from home (ICC = 0.058) and hypertension (ICC = 0.043) (Table 3). Therefore, to achieve the same statistical power for an individual randomized trial as would be obtained by a CRT, the number of subjects enrolled in the study should be multiplied by 2.68 and 2.25, respectively for a mean cluster size of 30. As shown in Table 4, ICC estimates of outcome variables in the CP group ranged from 0.001 for in-hospital mortality and disease severity at discharge to 0.069 for appropriateness of the stay (AOS) and median value and interquartile range for outcome measures ICCs was 0.006 [0.001-0.065]. The estimates for appropriateness of the stay, length of stay were 0.069 [0.003-0.155] and, 0.063 [0.007-0.311], respectively.

ICC estimates of outcome variables in the UC group ranged from 0.001 for in-hospital mortality to 0.203 for appropriateness of the stay (AOS) and median value and interquartile range for ICCs of outcome variables was 0.033 [0.003-0.187]. The estimates for appropriateness of the stay and disease severity at discharge were 0.203 [0.059-0.436], and 0.182 [0.062-0.554], respectively.

## Discussion

In the present study, ICCs and their associated 95% confidence intervals were calculated for clinical and patient-related outcome variables based on the results of an Italian multi-center cluster randomized trial of heart failure.

In recent years, the need to have published ICCs from different CRTs was put forward to help planning future studies [29-31]. Also several studies reported estimates of ICCs for various outcomes and for different treatment modalities [18,29,32]. However, this is the first study to present ICCs for a cluster randomized trial of care pathway. In addition, most of the studies have reported ICCs obtained in the setting of primary or residential care [19,22,29,33,34]. This study provides ICCs for a specific in-hospital treatment. Moreover, while some studies reported ICCs for many cardiovascular interventions [18,22,25], none of them reported ICCs for outcomes such as in-hospital mortality or length of hospital stay to determine effectiveness and efficiency of heart failure treatment. Although a well-designed CRT was conducted with the aim to show the effect of pharmacological treatment on heart failure [35,36], ICC estimates are still lacking. To our best knowledge, this is also the first study to present ICCs for multiple outcome variables in an effort to evaluate effectiveness and efficiency of in-hospital treatment of heart failure.

We hope that our estimates will be helpful in designing a clinical experiment not only for CPs but also for any clustered RT in heart failure patients. However, we believe that there is one critical point that should be considered by any researcher who would intend to use these estimates. We often observed less variation in different clusters in the CPs compared to the UC in many cases because all of the hospitals in the care pathway group used the same protocol and they were informed and trained in the same way; thus, ICC estimates tended to be lower, which implies the presence of larger within- cluster variance. The variance within clusters may be reduced by adjusting the subject-specific covariates in order to improve the accuracy of the ICC estimation.

It has been reported that ICCs were usually between 0.01 and 0.02 in human studies [34] and the Minnesota Heart Health Program Trial, the largest community trial for prevention of coronary heart disease to date, found ICCs which generally ranged between 0.002-0.012. In our study, the range of ICCs was wider (Tables 3 and 4).

We conducted a literature search to identify ICC estimates of variables similar to those in our study to compare our results. Previously published ICC estimates were available only for hypertension. We observed a moderate dependency for hypertension and previous estimates were also rather low for hypertension.

In primary care, physician practices would be expected to be more independent and previous studies showed that the ICC estimates derived from secondary care were greater than those from primary care [37]. In contrast to other studies, we found greater ICCs for outcome variables such as LOS, AOS and cost, whereas the ICCs for baseline characteristics tended to be lower. High ICCs estimated for LOS, AOS and cost indicated that patients staying in the same hospital shared many common characteristics other than patients in other hospitals. In other words, for these outcomes, HF management was more likely to be influenced by the practice itself or to be related with physician’s practice style. A care pathway is performed to encourage physicians practice more consistently. If the intervention is successful, it is expected that ICC based on post-intervention data would be smaller than an ICC based on pre-intervention data [38]. Based on our data, ICC value for the disease severity at admission [ICC = 0.046] was higher than that of disease severity at discharge in the care pathway group [ICC = 0.000]. This result confirms the success of the intervention and also consistency of the ICC estimates.

Estimation of the effect size or minimally important effect of intervention is another important consideration for sample size calculation. It is usually obtained from the published literature. Since a CRT design was used in the current study, our results may be helpful for estimating the effect size in future randomized trials of in-hospital treatment of heart failure.

Adjustment for the data variations in cluster was advised in many publications with regard to baseline covariates and factors such as age and gender based on the idea that different distributions across clusters could have an effect on ICC estimations. Several methods for adjustment have been discussed [39,40]. However, we did not make any adjustment because our clusters were very similar to each other with respect to baseline characteristics (Table 1). The results of this study were derived from patients between 50 and 99 years of age and may be extrapolated to other patients older than 50 years of age.

Many estimators have been proposed for binary outcomes [41] and point estimation by ANOVA is most commonly used for calculating ICC for continuous data [42]. Also there are several published studies which reported ICCs and each of them reported their ICC values in their own way. To ensure that all of the necessary information was presented in our report, we provided compressive information for reporting ICCs by using a framework reported by Campbell et al. in 2004 [37].

Since sample size estimation is a key element of a clinical trial, many new techniques have been introduced for cluster randomized clinical trials in the recent publications. Campbell et al. was performed a study to generate empirical estimates of ICCs and to explore factors which may affect their magnitude [38]. Furthermore, Turner et al. provided Bayesian methods of analysis for cluster randomized trials with binary outcome data [43]. Campbell et al. presented the development of a sample size calculator for cluster randomized trials and described strategies for increasing the sample size [44]. This calculator allows the investigator to trade-off the options for achieving the appropriate sample size. Most cluster randomized trials have unequal cluster sizes. Moreover, Eldridge et al. (2006) [45] showed how coefficient of variation of a cluster size can be used to deduce the possible effect of unequal cluster sizes for various types of analyses and both continuous and binary outcomes. Their simple formula provides a good estimate of sample size requirements for trials analysed using cluster-level analyses weighted by cluster size and a conservative estimate for other types of analyses. Furthermore, Sample size formulae for CRCTs with a fixed number of clusters for both continues and binary outcomes were systematically outlined by Hemming et al. [46]. In addition that Rotondi and Donner (2012) provided an evidence-based approach [47] and authored an R package to facilitate sample size estimation in this design (CRT Size). In this approach, sample size for clustered randomized trials was estimated taking into account the role of the planned trial on a future meta-analysis [48].

In the cluster randomized trials, the design effects are calculated using the cluster sizes from the existing data set and anyone planning a trial will need to calculate their sample size on their own. Thus, we did not report the design effects because it would not be useful for researchers planning their own trials.

There are some potential limitations of this study. The first limitation is the absence of any published ICC values for any care pathway or in-hospital treatment of heart failure. We have only been able to compare our results with ICCs estimated for different kinds of treatments. Second limitation of this study was that the data were collected between 2003 and 2004, to the best of our knowledge; this is the latest multicenter cluster randomized trial to show the effectiveness of CPs on the heart failure treatment. Also, no ICC values have been reported in literature for hospital treatment of heart failure up to date. But there is no evidence about the timeless of the material. So the readers should be aware of that changes in the treatment methodology can affect the ICC estimations and one should consider limitations of this study while using the ICC estimation of the present study.

## Conclusions

Although there are many previously published reports on ICC estimates, studies vary with respect to setting and outcome variables of interest. Also, it is often difficult to obtain a reliable ICC estimate applicable for a proposed study [17]. Therefore, specific ICC estimates are needed to design a CRT for heart failure treatment. In the present study, ICCs for multiple outcomes were reported with the aim to help facilitate the design process of future cluster randomized trials, particularly for therapeutic interventions for hospitalized heart failure patients.

## Abbreviations

AOS: Appropriateness of the stay; HF: Heart failure; CP: Care pathways; CRT: Cluster randomized clinical trial; ICC: Intraclass correlation coefficient; LOS: Length of in-hospital stay; NYHA: New York Heart Association; RCT: Randomized controlled trial; UC: Usual care.

## Competing interests

The authors declare that they have no competing interests.

## Authors’ contributions

SK, KV and MP participated in designing the study and MP provided the data from a previously conducted CP trial. SK conceived of the study and wrote the first draft of the paper. MP and KV helped writing the final manuscript and contributed to discussion of the results. All authors read and approved the final manuscript.

## Authors’ information

MP is the President of the European Pathway Association, E-P-A [http://www.E-P-A.org], KV is the Secretary General of E-P-A.

## Pre-publication history

The pre-publication history for this paper can be accessed here:

http://www.biomedcentral.com/1472-6963/14/84/prepub

## References

[B1] Ukoumunne OC Gulliford MC Chinn S Sterne JAC Burney PGJ Donner A Evaluation of health interventions at area and organization level BMJ 1999 14 376 379 10.1136/bmj.319.7206.376 10435968 PMC1126996

[B2] Donner A Klar N Cluster randomisation trials in epidemiology: theory and application J Stat Plan Interference 1994 14 37 56 10.1016/0378-3758(94)90188-0

[B3] Donner A Sample size requirements for strafied cluster randomisation designs Stat Med 1992 14 6 743 750 10.1002/sim.4780110605 1594813

[B4] Donner A Birkett N Buck C Randomization by cluster sample size requirements and analysis Am J Epidemiol 1981 14 906 914 7315838 10.1093/oxfordjournals.aje.a113261

[B5] Mcghee G Murphy E Research on reducing hospitalizations in patients with chronic heart failure Home Healthc Nurse 2010 14 335 340 10.1097/NHH.0b013e3181df5e30

[B6] Cowie MR Fox KF Wood DA Metcalfe C Thompson SG Coats AJ Poole-Wilson PA Sutton GC **Hospitalization of patients with heart failure. A population-based study** Eur Hearth J 2002 14 877 885 10.1053/euhj.2001.2973 12042009

[B7] American Heart Association. Heart Disease and Stroke Statistics: 2010 Update A report from the American heart association statistics committee and stroke statistics doi:10.1161/CIRCULATIONAHA.109.192667 Published online ahead of print 17 December 2009

[B8] Mariotto AB Yabroff KR Shao Y Feuer EJ Brow ML Projections of the cost of cancer care in the United States 2010–2020 J Nat Cancer Inst 2011 14 117 128 10.1093/jnci/djq495 21228314 PMC3107566

[B9] Vanhaecht K **The impact of care pathways on the organization of care processes** Acco, Leuven 2007 157

[B10] Campbell H Hotchkiss R Bradshaw N Porteous M Integrated care pathways BMJ 1998 14 133 137 10.1136/bmj.316.7125.133 9462322 PMC2665398

[B11] Panella M Marchisio S Di Stanislao F **Reducing clinical variations with clinical pathways: do pathways work**? Int J Qual Health Care 2003 14 509 521 10.1093/intqhc/mzg057 14660534

[B12] Azad N Molnar F Byszewski A Lessons learned from a multidisciplinary heart failure clinic for older women: a randomised controlled trial Age Ageing 2008 14 282 287 10.1093/ageing/afn013 18285347

[B13] Panella M Marchisio S Demarchi ML Manzoli L Stanislao FD Reduced in-hospital mortality for heart failure with clinical pathways: the results of a cluster randomised controlled trial Qual Saf Health Care 2009 14 369 373 10.1136/qshc.2008.026559 19812099

[B14] Rauh RA Schwabauer RN Enger EL Moran JF A Community hospital-based congestive hearth failure program: Impact on length of stay, admission and readmission rates and cost Am J Manag Care 1999 14 37 43 10345965

[B15] Philbin E Thomas AR Lindenmuth NW Ulrich K McCall M Jenkins PL The results of a randomized trial of quality improvement intervention in the care of patients with heart failure AM J Med 2000 14 443 449 10.1016/S0002-9343(00)00544-1 11042232

[B16] Panella M Vanhaecht K Is there still need for confusion about pathways? Int J Care Pathways 2010 14 1 3 10.1258/jicp.2010.010008

[B17] Murray DM Catellier DJ Hannan PJ Treuth MS Stevens J Schmitz KH Rice JC Conway TR School level intraclass correlation for physical activity in adolescent girls Med Sci Sports Exerc 2004 14 876 882 15126724 10.1249/01.mss.0000126806.72453.1c PMC2040294

[B18] Hannan PJ Murray DM Jacobs DR McGovern PG Parameters to aid in the design and analysis of community trials: intraclass correlation from the Minesota heart health program Epidemiology 1994 14 88 95 10.1097/00001648-199401000-00013 8117787

[B19] Cosby R Howard M Kaczorowski J Willian AR Sellors JW Randomizing patients by family practice: sample size estimation, intracluster correlation and data analysis Fam Pract 2003 14 77 82 10.1093/fampra/20.1.77 12509376

[B20] Adams G Gulliford MC Ukoumunne OC Eldridge S Chinn S Camphell MJ Patterns of intra-cluster correlation from primary care research to inform study design and analysis J Clin Epidemiol 2004 14 785 794 10.1016/j.jclinepi.2003.12.013 15485730

[B21] Elley CR Kerse N Chondros P Robinson E Intraclass correlation coefficient from three cluster randomised controlled trials in primary and residential health care Aust N Z J Public Health 2005 14 461 467 10.1111/j.1467-842X.2005.tb00227.x 16255449

[B22] Parker DR Evangelou E Eaton CB Intraclass correlation coefficients for cluster randomized trials in primary care: the cholesterol education and research trial [CEART] Contemp Clin Trials 2005 14 260 267 10.1016/j.cct.2005.01.002 15837446

[B23] Panella M Marchisio S Gardini A Di Stanislao F A cluster randomized controlled trial of a clinical pathway for hospital treatment of heart failure: study design and population BMC Health Serv Res 2007 14 179 185 10.1186/1472-6963-7-179 17986361 PMC2204000

[B24] Donner A Klar N Design and analysis of cluster randomization trials in health research 2000 London: Arnold

[B25] Smith CAB On the estimation of intraclass correlation Ann Hum Genet 1956 14 363 373

[B26] Golstein H Browne H Rasbash J Partitioning variation in multilevel models Underst Stat 2002 14 4 223 231 10.1207/S15328031US0104_02

[B27] Ukoumunne OC A comparison of confidence interval methods for the intraclass correlation coefficient in cluster randomized trials Stat Med 2002 14 3757 3774 10.1002/sim.1330 12483765

[B28] Kerryand SM Bland JM Statistics notes: the intracluster correlation coefficient in cluster randomisation BMJ 1998 14 1455 1460 9572764 10.1136/bmj.316.7142.1455 PMC1113123

[B29] Ukoumunne OC Gulliford MC Chinn S Sterne JAC Burney PGJ Methods for evaluating area-wide and organisation-based interventions in health and health care: a systematic revi*ew* Health Technol Assess 1999 14 5 iii 92 10982317

[B30] Murray DM Bliststein JL Methods to reduce the impact of intraclass correlation in group-randomized trials Eval Rev 2003 14 79 103 10.1177/0193841X02239019 12568061

[B31] Killip S Mahfounf Z Pearche K What is an intraclass correlation coefficient? Crucial concepts for primary care researchers Ann Fam Med 2004 14 204 208 10.1370/afm.141 15209195 PMC1466680

[B32] Gulliford MC Ukoumunne OC Chinn S Components of variance and intraclass correlations for the design of community-based surveys and intervention studies: data from the health survey for England 1994 Am J Epidemiol 1999 14 876 883 10.1093/oxfordjournals.aje.a009904 10221325

[B33] Smeeth L Ng S Intraclass correlation coefficients for cluster randomized trials in primary care: data from MRC trial of the assessment and management of older people in community Control Clin trials 2002 14 409 421 10.1016/S0197-2456(02)00208-8 12161083

[B34] Murray DM Rooney BL Hannan PJ Peterson AV Ary DV Biglan A Botvin GJ Evans RI Flay BR Futterman R Intraclass correlation among common measures of adolescent smoking Am J Epidemiol 1992 14 1038 1050 10.1093/oxfordjournals.aje.a117194 7985652

[B35] Lowrie R Mair FS Greenlaw N Forsyth P Jhund PS McConnachie A Rae B McMurray JJ Heart Failure Optimal Outcomes from Pharmacy Study (HOOPS) Investigators. Pharmacist intervention in primary care to improve outcomes in patients with left ventricular systolic dysfunction Eur Heart J 2012 14 3 314 324 10.1093/eurheartj/ehr433 22083873

[B36] Lowrie R Mair FS Greenlaw N Forsyth P McConnachie A Richardson J Khan N Morrison D Messow CM Rae B McMurray JJ The Heart failure and Optimal Outcomes from Pharmacy Study (HOOPS): rationale, design, and baseline characteristics Eur J Heart Fail 2011 14 8 917 924 10.1093/eurjhf/hfr083 21791543

[B37] Campbell MK Grimshaw JM Elbourne DR Intracluster correlation coefficients in cluster randomized trials: empirical insights into how should they be reported BMC Med Res Methodol 2004 14 4 9 10.1186/1471-2288-4-9 PMC415547 15115554

[B38] Campbell M Grimshaw J Steen N Sample size calculations for cluster randomised trials. Changing professional practice in Europe group [EU BIOMED II concerted action] J Health Serv Res Policy 2000 14 1 12 16 10787581 10.1177/135581960000500105

[B39] West BT Welch KB Galecki AT Linear mixed models: a practical guide using statistical software 2007 Chapman & Hall: Boca Raton

[B40] Yelland LN Salter AB Ryan P Laurence CO Adjusted intraclass correlation coefficients for binary data: methods and estimates from a cluster-randomized trial in primary care Clin Trials 2011 14 48 58 10.1177/1740774510392256 21335589

[B41] Zou G Donner A Confidence interval estimation of the intraclass correlation coefficient for binary data outcome data Biometrics 2004 14 807 811 10.1111/j.0006-341X.2004.00232.x 15339305

[B42] Donner A Koval J Design consideration in the estimation of the intraclass correlation Ann Hum Genet 1982 14 271 277 10.1111/j.1469-1809.1982.tb00718.x 7125598

[B43] Turner RM Omar RZ Thompson SG Bayesian methods of analysis for cluster randomized trials with binary outcome data Stat Med 2001 14 453 472 10.1002/1097-0258(20010215)20:3<453::AID-SIM803>3.0.CO;2-L 11180313

[B44] Campbell MK Thomson S Ramsay CR MacLennan GS Grimshaw JM Sample size calculator for cluster randomised trials Comput Biol Med 2004 14 2 113 125 10.1016/S0010-4825(03)00039-8 14972631

[B45] Eldridge S Kerry S Ashby D Sample size for cluster randomized trials: effect of coefficient of variation of cluster size and analysis method Am J Epidemiol 2006 14 1292 1300 10.1093/ije/dyl129 16943232

[B46] Hemming K Girling AJ Sitch AJ Marsh J Lilford RJ Sample size calculations for cluster randomised controlled trials with a fixed number of clusters BMC Med Res Methodol 2011 14 11 102 106 21718530 10.1186/1471-2288-11-102 PMC3149598

[B47] Rotondi M Donner A Sample size estimation in cluster randomized trials: an evidence-based perspective Comput Stat Data Anal 2012 14 5 1174 1187 10.1016/j.csda.2010.12.010

[B48] Rotondi M CRT size: sample size estimation for cluster randomized trials 2012 http://cran.r-project.org/web/packages/CRTSize/CRTSize.pdf

